# Process development for high-titer production of adenovirus devoid of replication-competent particles in suspension-adapted complementing A549 cell culture

**DOI:** 10.1186/s12896-025-01051-8

**Published:** 2025-11-03

**Authors:** Chun Fang Shen, Elodie Burney, Rénald Gilbert, Sonia Tremblay, Martin Loignon

**Affiliations:** Human Health Therapeutics Research Centre, National Research Council of Canada, Montreal, Canada

**Keywords:** Adenovirus, A549 cells, SF-BMAdR cells, Culture media

## Abstract

Adenovirus is one of the most attractive viral vectors for therapeutic vaccines and gene therapy with the caveat that replication-competent adenoviruses (RCA) can be produced. To remediate this problem, engineered A-549 adenoviral vector complementing cells (SF-BMAdR cells) were previously generated by our organization for the production of E1-deleted adenoviral vectors without RCA. However, the production process remained to be improved for high titer production and scalability, as cost-effective and scalable biomanufacturing processes are critical for commercializing adenovirus-based vaccines and gene therapy. In this study, we first explored the potential of batch and fed-batch culture to increase maximum cell density and virus productivity by evaluating four different commercially available serum-free media and their combinations, and several feeds. A mixture (1:1) of two culture media improved the maximum cell density from 2.8 × 10^6^ cells/mL obtained in the current batch culture to 4.2 × 10^6^ cells/mL, and increased the virus productivity by 70% at a titer of 1.5 × 10^10^ vp/mL. The fed-batch culture process, however, did not yield a significant improvement in either the maximum cell density or virus productivity. In contrast, batch culture with one medium replacement not only increased the cell growth but also resulted in an additional 70% improvement in the virus productivity at 2.6 × 10^10^ vp/mL. The virus productivity was further increased to 6.3 × 10^10^ vp/mL in a 3 L bioreactor perfusion culture infected at 7.0 × 10^6^ cells/mL. This titer is 7.5 folds of the titer obtained in the current process. This study demonstrated the potential for a drastic improvement in the productivity of RCA-free adenovirus in the SF-BMAdR culture process. Furthermore, various processes developed fulfill different operational needs in manufacturing of RCA-free adenovirus to meet the increasing demands for therapeutic vaccines and gene therapy.

## Introduction

Over the past few decades, viral vectors have been improved to satisfy regulatory requirements to serve increasing medical applications. Adenovirus is one of the most attractive viral vectors for therapeutic vaccines and gene therapy because of its well-defined biology and characteristics and the availability of platforms for rapid production. Among the advantages of adenoviral vectors is their favorable safety profile, and the relatively large cargo that can be carried and expressed to stimulate a robust cellular and humoral immune response in vaccine applications. Such qualities and others have led to adenoviruses being the most used gene transfer vectors in experimental therapies and therapeutic vaccines as evidenced by the approval of several adenoviral vector-based vaccines such as COVID 19, Ebola and cancer [1–4].

Adenoviral vectors most used in vaccine production and gene therapy are non-replicating (or replication-deficient) whereby the genome is deleted in the E1 region often in combination with the E3 region to provide space for alternate gene expression cassettes. Due to the E1 deficiency in adenoviral vectors, special producer cells that trans-complement the E1 function are essential to produce replication-incompetent adenoviruses. Several cell lines for manufacturing such vectors have been described [5], of which HEK293 is the most common and available to the scientific community [6]. However, the disadvantage of the HEK293 cell line is the potential for RCA contamination within the viral preparation. This can occur because HEK293 cells have an extensive sequence homology with the engineered adenoviral vectors, allowing for homologous recombination events to occur during the production process. This is problematic for large-scale viral production and clinical applications. The fundamental concern for the presence of RCA in viral products is safety as RCA may replicate in an uncontrolled manner in the patient. The FDA has established that the presence of RCA in replication-defective adenoviral vectors must be lower than 1 RCA in 3 × 10^10^ viral particles (vp). To avoid/prevent the occurrence of RCAs during adenovirus production, some effort has been put into the generation of alternative cell lines by eliminating any sequence overlap between viral sequences in the E1-deleted virus and those present in the cell line [7] and were reviewed by Kovesdi and Hedley [5]. Even though a wide array of producer cell lines (such as HEK293, PER.C6, N52.E6) have been developed to produce adenoviral vectors throughout the last four decades, there are still only a few cell lines approved for cGMP production of replication-defective adenovirus type 5.

We have engineered an adenoviral vector complementing cell line (SF-BMAdR) which was derived from A-549 cells and stably expresses the minimal E1A and E1B genes [8]. An established SF-BMAdR-281 clone has been tested in different cell culture media, adapted to grow in suspension culture in serum-free commercially available media, and to produce vectors at a competitive titer of 8 × 10^9^ vp/mL. The vectors produced by SF-BMAdR cells were free of RCA or below a detection limit of 1 RCA in 5.3 × 10^10^ VP [8, 9]. Additionally, the clone was also designed to facilitate the production of an adenoviral vector encoding transgene for cytotoxic gene products. With increasing demands on the usage of these cells for the production of adenoviral vectors for therapeutic vaccines and gene therapy, the development of a high-titer process is required to reduce the production cost of mass material required for clinical trials. In addition, the price per dose is an important determining factor of economic viability when adenovirus is used for vaccine applications. Also, the trend towards higher doses for viral vector-based therapies intensifies the need for greater cost-efficiency and economy of scale in clinical development and manufacturing. The current study aimed to develop cell culture processes to improve the productivity of adenovirus type 5 vector.

## Materials and methods

### >Cell lines, media, and virus

SF-BMAdR-281 clone was maintained in Pro293s-CDM medium (the reference media, referred to as Pro293s; Lonza, Walkersville, MD) at 25 mL working culture volume in 125 mL polycarbonate plastic shake flasks (Corning, NY) at 37 °C and 5% CO_2_ with agitation of 120 rpm in a humidified incubator. They were subcultured to 2 or 2.5 × 10^5^ cells/mL, 3 times a week. The culture was sampled regularly for cell counts and other analyses as needed. The clone was discarded after 2 months in culture.

There is no commercial serum-free media specifically designed to support the growth of the suspension adapted SF-BMAdR-281 clone or A549 cells. The media supporting 293 cell growth and adenovirus production were used in this study. They were Pro293s, HyCell™ TransFx-H (referred to as HyCell; Cytiva, Logan, UT), BalanCD HEK293 (referred to as BalanCD; FUJIFILM Irvine Scientific, Santa Ana, CA), and HEK TF (Sartorius, Bielefeld, Germany). Feeds included Cell Boost 5 (Cytiva), HEK FS (Sartorius), yeast extract (Biospringer), Sheff-Vax ACF (Kerry Bio-Science, Norwich, NY), amino acids or their combination. The Cell Boost 5 (or CB5) feed was prepared at a concentration of 35 g/L according to the manufacturer’s instructions.

Adenovirus type 5 (Ad5-GFP) and the titer of viral stock used for culture infection were described before [10].

## Shake flask batch and fed-batch cultures

The suspension SF-BMAdR-281 clone was thawed and grown in Pro293s medium. The growth of the SF-BMAdR-281 clone in other media in batch culture was only evaluated after the clone was maintained in each of the selected media for at least two weeks. In a separate set of experiments, when cell density in batch cultures reached about 1, 2, and 3 × 10^6^ cells/mL, the cultures were infected with Ad5-GFP at a multiplicity of infection (MOI) of 10 IVP/cell. The infected cultures were sampled at 48 and 72 hours post-infection for cell count and metabolite analysis (if needed) and stored at −80 °C until Ad5-GFP quantification by HPLC. All shake flask cultures in this and following sections were conducted in duplicate.

Two top-performance media (Pro293s and HyCell) supporting high cell density and virus productivity were selected after the media evaluation and employed as basal media for the development of the fed-batch culture process. The cell growth in fed-batch culture without virus infection was conducted by feeding the culture with 5% CB5, 5% HEK FS, 2 g/L yeast extract, 1 g/L Sheff-vax-ACF, a mixture of amino acids or a combination of feed and individual components to measure their potency to increase maximum cell density. Some of the above feeds were also added to cultures infected at 1 and 2 × 10^6^ cells/mL after virus infections to measure their impact on the virus productivity.

## Shake flask batch culture with medium replacement(s)

SF-BMAdR-281 clone was grown in Pro293s + 5% CB5, HyCell, or a mixture of 50% Pro293s + 50% HyCell to cell densities of 1, 2 and 3 × 10^6^ cells/mL respectively. The cells were then centrifuged at 300 g for 5 minutes at room temperature. According to the experimental design in Table 1, the cell pellets were resuspended in their respective fresh media to obtain final densities of 1, 2, and 3 × 10^6^ cells/mL before infection with Ad5-GFP. In addition, some shake flask cultures with SF-BMAdR-281 clones grown in a mixture of 50% Pro293s + 50% HyCell were subjected to 2 medium replacements when the culture reached a respective density of ~2.5 × 10^6^ cells/mL and 5 × 10^6^ cells/mL during the cell growth phase, in order to reach desired higher cell densities of 6 to 8 × 10^6^ cells/mL. At the desired cell density, the cells were infected with Ad5-GFP followed by 3 to 4 medium replacements post the virus infection (to mimic the perfusion culture process). The infected cultures were sampled at 48 and 72 hours post-infection for cell count, metabolite analyses, and virus titration.

**Table 1:** Experimental design for evaluating the effect of culture media and cell density on virus productivity

Media used in cell growth phase	Media used in the virus production phase			Cell density at infection (E + 06 cells/mL)		
	Pro293s	HyCell	50% Pro293s + 50% HyCell	1	2	3
Pro293s + 5%CB5	+			+	+	+
Pro293s + 5%CB5		+		+	+	+
HyCell	+			+	+	+
HyCell		+		+	+	+
50% Pro293s + 50% HyCell			+	+	+	+

Note: + indicates the culture condition was tested

## Bioreactor culture

Bioreactor culture in batch mode: Bioreactor experiments were conducted in a BioFlo 320 system (Eppendorf), fitted with a 5 L BioBLU 3c vessel with a final working volume of 3 L. The controls of temperature (37°C), agitation (125 rpm), dissolved oxygen (DO, 40%), and pH (7.15 ± 0.05) were previously described by Shen et al. [11]. The SF-BMAdR-281 clone grown in a 2 L shake flask was used to inoculate the bioreactor at a density between 0.20 to 0.30 × 10^6^ cells/mL in a media mixture of 50% Pro293s and 50% HyCell. After the bioreactor inoculation, a small portion of the culture was sampled from the bioreactor and transferred into a shake flask as an internal control. After reaching 1.0 to 1.5 × 10^6^ cells/mL, cells in both the bioreactor and control cultures were infected with the Ad5-GFP stock at a MOI of 10. Samples from both the bioreactor and control shake flask cultures were collected regularly for analysis.

Bioreactor culture in perfusion mode: The perfusion culture was conducted in a BioFlo 320 system with a 5 L BioBLU 3c vessel equipped with an ATF (Xcell® ATF2, Repligen) as a cell retention device. The bioreactor was inoculated according to the procedure described in the previous section. After reaching the targeted cell density of about 1.5 × 10^6^ cells/mL, a perfusion process was started at a rate of 1 volume of medium per bioreactor working volume per day (VVD). The perfusion rate was regulated during the culture process to meet the needs of cell growth and virus production. Cells were infected at a density of 7.0 × 10^6^ cells/mL at MOI 10. A perfusion rate of 3.5 VVD was maintained for 32 hours post the virus infection. A control shake flask culture with multiple medium replacements during the cell growth and post virus infection was conducted to have similar conditions to the perfusion bioreactor culture. Samples from the both bioreactor and control shake flask cultures were collected regularly for analysis.

## Analytical methods

Accumax solution (Innovative Cell Technologies, Inc.) was mixed 1:2 with culture sample in a 1.5 mL vial and then incubated at 37 °C under agitation for 30 min. Cell counts and viability were assessed with an automated cell counter (CedexHires Automated Cell Counter) or using a hemacytometer and erythrosine B. Glucose, lactate dehydrogenase (LDH), lactate, and ammonia were analyzed by Cedex Bio Analyzer (Roche CustomBiotech). Total virus particle titers were assayed by HPLC as described in Klyushnichenko et al. [12].

## Statistical analysis

The means from two groups were compared using a Student t-test (The Excel Analysis ToolPak). Some means were also analyzed using an analysis of variance (ANOVA) followed by a Fishers (LSD) test. Statistical significance was set at *p* < 0.05.

## Results and discussion

**Fig. 1:**
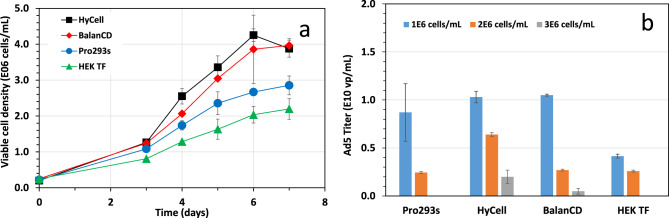
**a:** Growth profile of SF-BMAdR cells in batch cultures with 4 different commercial culture media;**b**: production of adenovirus 5 in batch cultures growing with 4 respective media and infected at 3 different cell densities. Bars represent the mean of duplicate cultures ± standard deviation. The virus productivity in the cultures infected at 1E6 cells/mL was not statistically significant (*p* = 0.07)

Growth of SF-BMAdR cells and production of Ad5-GFP in batch culture with different culture media: Figure 1a shows the growth profile of SF-BMAdR cells in batch cultures respectively utilizing the four commercial serum-free media described in the section of materials and methods. The SF-BMAdR cells grew to a maximum viable cell density of 2.8 × 10^6^ cells/mL in Pro293s. This chemically defined Pro293s was tested in an earlier study [8] and used as the reference medium in this experiment. Both HyCell and BalanCD supported the cell growth up to a respective density of 4.2 × 10^6^ and 4.0 × 10^6^ cells/mL, however, significant cell aggregates were formed in the culture using BalanCD. Finally, in HEK TF medium, the cells reached a maximum density of 2.2 × 10^6^ cells/mL coupled with a longer cell doubling time of 40 hours. Cell viability in all cultures was higher than 98% during the process of batch culture.

Both HyCell and BalanCD supported the growth of SF-BMAdR-281 clone reasonably well, considering these media were not tailored for SF-BMAdR-281 clone. Although HEK TF medium can support a robust growth of a HEK293 clone up to 8 × 10^6^ cells/mL [13], the SF-BMAdR-281 clone grew poorly in this medium. These results suggest that the nutritional requirement could vary dramatically for the growth of individual cell lines. Some media such as HyCell might be formulated with a broad spectrum of nutrients, which therefore is more versatile in supporting the growth of different cell lines.

The production of Ad5-GFP was assessed for the SF-BMAdR-281 cultures grown in different media and infected at various cell densities. HPLC analysis of infected cultures revealed that higher Ad5 titers were detected in the cultures sampled at 48 hpi than the ones at 72 hpi. Figure 1b shows that the culture infected at 1 × 10^6^ cells/mL produced the best titer of the virus in all of the 4 culture media tested. The highest titer ranged from 8.5 × 10^9^ to 1.2 × 10^10^ vp/mL, except the virus production was 4.1 × 10^9^ vp/mL in the culture using HEK TF. The cell-specific productivity for the culture cultivated in Pro293s, HyCell, BalanCD and HEK TF and infected at 1 × 10^6^ cells/mL was 6600, 8879, 6000 and 4600 vp/cell respectively. The virus productivity in the SF-BMAdR-281 culture however dropped rapidly when the SF-BMAdR-281 culture was infected at increased cell density such as ≥ 2 ×10^6^ cells/mL, suggesting the decline in adenovirus production in the cultures infected at high cell density was due to reduced cell-specific productivity. In this case, the breakpoint related to the specific production drop was at 1 × 10^6^ cells/mL. This limitation has been referred to as the “cell density effect” [14]. Nutrient limitations and/or accumulation of inhibitory metabolites generally contribute to the cell density effect. However, due to the complexity of culture media formulation and metabolites secreted from cells, the exact nutrient component(s) or inhibitory metabolite(s) remains largely unknown [15]. As the cells cultured in Pro293s and HEK TF were not able to reach a cell density of 3 × 10^6^ cells/mL, no virus production was carried out at this cell density for these two media.

The virus productivity of the SF-BMAdR-281 cells were compared with other 3 cell lines which have been generated and/or used for production of replication-defective adenoviral vectors. Table 2 shows that the virus productivity of the SF-BMAdR-281 cells is in a similar range of titers obtained in other cell lines (assuming 5% to 10% of virus produced by 293FLP is infectious). However, 293FLP and N52.E6 are adherent cells, which are not as easy as suspension cells in scale up for large scale manufacturing. Although the virus productivity of PER.C6 is at the high end, owing to licensing costs, the general application of this cell line is strictly limited. We also found that 1.2 × 10^10^ vp/mL obtained in the SF-BMAdR-281 culture using HyCell and infected at 1 × 10^6^ cells/mL is twice the titer (5.0 × 10^9^ vp/mL) produced by HEK293 cells cultivated and infected under the same condition.

**Table 2:** Comparison of virus productivity among different cell lines used for production of replication-defective adenoviral vectors

Cell lines	Type of cell culture	Cell specific virus productivity	Volumetric virus productivity	References
BMAdR	Suspension	8.8 × 10^3^ vp/cell	1.2 × 10^10^ vp/mL	This study
293FLP	Adherent	1 ~ 2 × 10^3^ pfu/cell	2 ~ 5 × 10^8^ pfu/mL	[16]
N52.E6	Adherent	1.0 ~ 2.6 × 10^4^ vp/cell		[17]
PER.C6	Suspension/Adherent	2 ~ 4 ×10^4^ vp/cell	8 ×10^10^ vp/mL	[18]

After taking into consideration of the culture media supporting cell growth and virus productivity and also culture quality, Pro293s and HyCell were selected for further studies on the development of fed-batch and other processes.

**Fig. 2:**
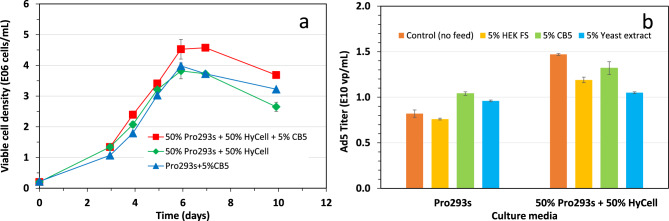
**a**: Improvement of cell growth by supplementing the culture with 5% cell boost 5 or mixing two media. The maximum viable cell obtained in the 3 cultures was not significantly different (*p* = 0.15); **b**: effect of feeding commercial feeds on the virus productivity in the culture infected at a cell density of 1 × 10^6^ cells/mL. Bars represent the mean of duplicate cultures ± standard deviation

Development of a fed-batch process for improvement of cell growth and virus production: The SF-BMAdR-281 clone respectively cultured in Pro293s and HyCell media was supplemented with various feeds such as 5% CB5, 5% HEK FS, 2 g/L yeast extract, 1 g/L Sheff-vax-ACF, a mixture of amino acids or a combination of various feeds through a few sets of experiments. Although the maximum cell density was improved by each feed to various extents, the overall increment in the maximum cell density was not dramatic. A combination of different feeds did not show a profound additional improvement in the maximum cell density. As a result, we only presented data from the cultures fed with a simplified feed combination and showed a significant increase in the maximum cell density. As shown in Fig. 2a, the maximum cell density increased from 2.8 × 10^6^ cells/mL to 4.0 × 10^6^ cells/mL when the cells cultured in Pro293s were fed with 5% CB5. Interestingly, a maximum cell density of 3.8 × 10^6^ cells/mL was obtained when the cells were cultured in a mixture of 50% Pro293s and 50% HyCell. Feeding this culture with 5% CB5 increased the maximum cell density by 0.8 × 10^6^ cells/mL, reaching 4.6 × 10^6^ cells/mL. These feeding strategies set the basis for further studies on the improvement of adenovirus production.

The effect of feeding the SF-BMAdR-281 culture at the time of viral infection was also investigated. Data in Fig. 2b show there was up to 20% improvement in the virus productivity when the culture grown in Pro293s was infected at 1 × 10^6^ cells/mL and then fed with 5% CB5 or yeast extract. The virus productivity however declined instead of improving in the culture grown in the mixture of 50% Pro293s + 50% HyCell when the culture was fed. In addition, the feeding did not improve the virus productivity either when the culture was infected at higher cell density at 2 × 10^6^ cells/mL or higher (data not shown). These results show that either increasing cell density of SF-BMAdR-281 cultures at the infection or the feeding strategy does not significantly increase virus productivity. These results resonate with our previous observations in suspension Vero cell fed-batch cultures [19].

Fed-batch culture, where nutrients are added intermittently during the process, is a method most widely used to increase cell density and volumetric productivity in cell cultures, especially monoclonal antibodies in CHO cultures [20, 21], offering higher titers and productivity compared to traditional batch cultures. Indeed, the maximum cell density and volumetric productivity of baculovirus and adenovirus have been significantly improved in fed-batch processes respectively with Sf9 and HEK293 cells [10, 22]. However, a dramatic improvement in the maximum cell density could not be always achieved in fed-batch culture, and culture infected at a higher cell density does not constantly correlate with an increase in virus productivity. This is the case for SF-BMAdR-281 clone in this study and also for Vero cells [11]. In these cell cultures, the maximum cell density in fed-batch culture is speculated to be limited, at least in part, by inhibitory metabolites accumulated in the culture, as feeding extensive nutrients (including FBS) did not significantly improve the maximum cell density.

It appears more challenging to increase virus productivity in a fed-batch process significantly. This could be due to different nutritional requirements during the cell growth and virus production in a biphasic viral and vector production process. Sufficient residual nutrients like glucose and amino acids in the cultures infected at between 1 × 10^6^ and 2 × 10^6^ cells/mL during the virus production phase did not warrant an improved virus production. It is unclear whether the inhibitory metabolites are more deleterious to the viral infection or the productivity of some cell lines such as SF-BMAdR-281 and Vero cells. Therefore, the precise factors limiting virus productivity in fed-batch cultures remain to be identified, possibly explaining why the development of fed-batch cultures for virus production has only been reported sporadically [23, 24].

**Fig. 3:**
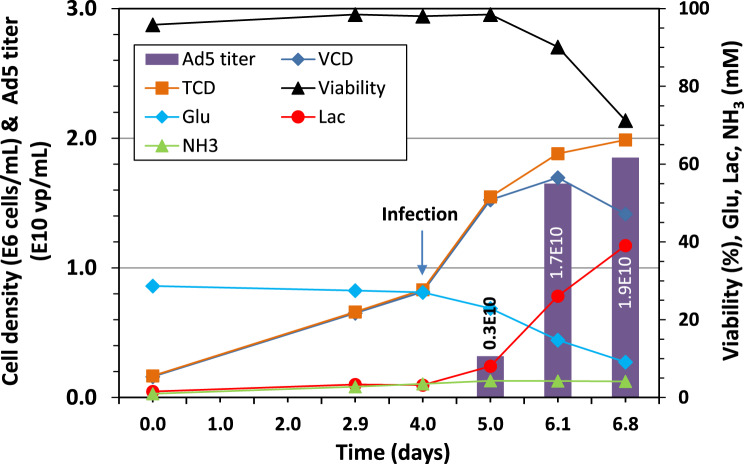
Scale-up of adenovirus 5 production in 3 L single use bioreactor batch culture using a mixture of 50% Pro293s and 50% HyCell media and infected at 0.8 × 10^6^ cells/mL without a medium replacement before the virus infection. The infected bioreactor culture was sampled at 24, 48 and 67 hpi for quantification of infectious and total viral particle concentration

Scale-up of Ad5-GFP production to 3 L bioreactor batch culture: Before developing other strategies to improve the virus productivity, the optimal culture condition obtained in shake flask culture for the virus production was scaled up to 3 L single use bioreactor to demonstrate the process scalability in 2 bioreactor runs. Figure 3 depicts the cell growth, glucose consumption, metabolite accumulation and also the virus production over the time course in one of the 3 L bioreactor culture run. The culture infected at 0.8 × 10^6^ cells/mL kept growing to 1.9 × 10^6^ cells/mL at 67 hours post infection (hpi). The cell viability decreased to 90% at 50 hpi, and continued to decline to 70% at the time of harvest (67 hpi). The glucose concentration remained more than 20 mM during the process while lactate and ammonia accumulated to 40 and 5 mM respectively.

A production of the virus at a titer of 3 × 10^9^ vp/mL was detected at 24 hpi, and the virus titer reached 1.7 × 10^10^ vp/mL at 48 hpi and 1.9 × 10^10^ vp/mL at 67 hpi. This titer is better than the one of 1.45 × 10^10^ vp/mL obtained in the shake flask culture (Fig 2b). The 3 L bioreactor batch culture was repeated and similar titers were obtained, respectively 1.97 × 10^10^ vp/mL at 48 hpi and 1.67 × 10^10^ vp/mL at 71 hpi. The highest titers obtained from the two bioreactor runs represented an average of the best cell-specific productivity of 10,349 ± 1133. These results suggest that productivity could be increased in better controlled culture conditions. Such an increase in productivity has been obtained in the production of other viruses in different platforms [10].

The samples from the first 3 L bioreactor culture were assayed for infectious titer which was 3.9 × 10^7^, 3.2 × 10^9^ and 1.6 × 10^9^ TCID50/mL respectively for the sample taken at 24, 48 and 67 hpi. This corresponds to a ratio of infectious to total virus particles of 1.3%, 18.5% and 8.3%. This result indicates that not only the productivity of total virus particle was affected by the harvest time, the ratio of infectious to total virus particles was also significantly impacted by the harvest time. Therefore, harvest time has a significant effect on the virus yield and also quality.

**Fig. 4:**
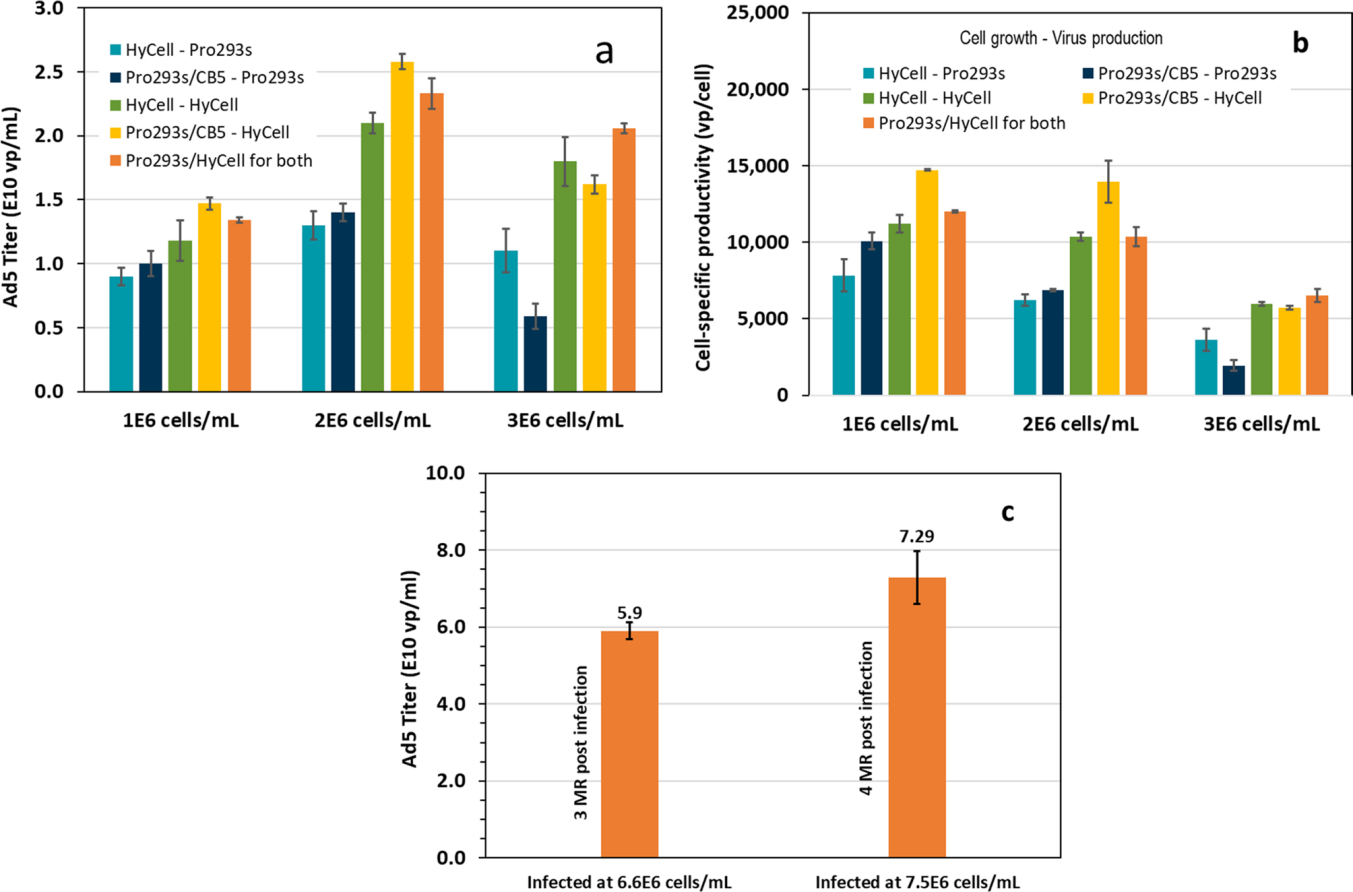
**a**: Volumetric productivity of adenovirus in batch cultures grown in 5 different culture media to a respective cell density of 1, 2 and 3 × 10^6^ cells/mL, followed by a medium replacement (MR), and virus production in the same or different medium (see Table 1 for experimental design); **b**: cell specific virus productivity; **c**: virus production in batch culture with 2 medium replacements prior to the virus infection, followed by multi medium replacements post the virus infection. Bars represent the mean of duplicate cultures ± standard deviation

*Virus production in batch culture with medium replacements.* So far, we have found that the volumetric virus productivity did not increase when the cultures were infected at a cell density higher than 1 × 10^6^ cells/mL in either batch or fed-batch culture. However, mixing two culture media improved the virus productivity up to 50% (Fig 2b). Therefore, a set of experiment including three factors (3 cell densities, two media used in cell growth phase and two media used during the virus production phase, as shown in Table 1) was conducted to determine if the virus productivity could be further improved by using different media for cell growth and for virus production respectively, and to examine the impact of cell density at infection. Data in Fig. 4a show that the increase in the virus productivity was marginal in the culture infected at 1 × 10^6^ cells/mL and with medium replacement when compared to the titer obtained in a similar culture infected at the same cell density without medium replacement. However, when the cell density at infection was increased from 1 to 2 × 10^6^ cells/mL, the titer increased up to 80% in the culture with medium replacement and using a mixture of 50%Pro293s + 50% HyCell during the virus production phase. The highest titer of 2.6 × 10^10^ vp/mL was obtained in the culture grown in Pro293s + 5% CB5 and infected at 2 × 10^6^ cells/mL, and the virus production phase was conducted in HyCell medium. This titer is significantly higher (*p* < 0.05) than the titer obtained under other culture conditions except the one using a media mixture for the both phases. This result illustrates that a combination of different culture media used in cell growth and virus production phase can significantly improve the virus productivity and suggests that growth and production phases have different nutritional requirements. In contrast, when the cells were grown in HyCell medium and the virus production phase was carried out in Pro293s medium, the virus productivity was the lowest, at 0.8 × 10^10^ and 1.3 × 10^10^ vp/mL, respectively, in the culture infected at a cell density of 1 or 2 × 10^6^ cells/mL. The virus productivity in the cultures using other media combinations was between 1.0 to 2.3 × 10^10^ vp/mL at the above cell densities infected. The virus productivity declined in all the cultures infected at 3 x 10^6^ cells/mL, and more significantly in the culture using Pro293s in the virus production phase.

The data in Fig. 4a indicate that Pro293s was a poor medium for the virus production when used during the virus production phase. The virus productivity in these cultures was low and did not increase significantly (*p* > 0.05) when the cell density at infection was increased from 1 to 2 × 10^6^ cells/mL. Instead, the virus productivity declined significantly (*p* = 0.015) when the culture was grown in Pro293s/CB5 and infected at 3x10^6^ cells/mL in Pro293s medium. In contrast, virus titer in the cultures using HyCell or 50% Pro293s + 50% HyCell in the virus production phase increased by 77% when the cell density at infection was increased from 1 to 2 × 10^6^ cells/mL, and did not decline dramatically in the culture infected at 3 × 10^6^ cells/mL.

The virus productivity in the culture using the mixture of 50% Pro293s + 50% HyCell during both the cell growth and virus production media was among the best, and less affected when the culture was infected at 3 × 10^6^ cells/mL. Therefore, the mixture of 50% Pro293s + 50% HyCell is a more robust medium and was selected for further process development.

The cell-specific productivity in Fig.4b reveals that when HyCell or HyCell/Pro293s was used as a medium in the virus production phase, the cell specific productivity was nearly maintained in the cultures infected at a cell density up to 2 x 10^6^ cells/mL. This again suggest the HyCell is a more robust medium for virus production.

A complete medium replacement prior to the virus infection provides fresh nutrients and also removes inhibitory metabolites. This operation particularly benefited the culture infected at higher cell density (such as 2 and 3 × 10^6^ cells/mL) with higher demands of nutrients and increased the virus productivity by up to 80%. While the titer improvement in the culture infected at 1 × 10^6^ cells/mL was not as significant when compared to the titer obtained in the respective culture infected without medium replacement. A probable explanation is that the nutrients needed for production were still available in the culture infected at 1 x 10^6^ cells/mL without medium replacement.

The data in Fig. 4a also reveal that the virus productivity was not only affected by the availability of nutrients in the culture media used during the virus production phase, but also influenced by the physiological state of cells grown in the culture media. The cells grown in Pro293s or Pro293s + 5% CB5 were more productive than the cells cultivated in HyCell or a mixture of 50% Pro293s + 50% HyCell. This shows that the physiological state of cell’s virus productivity could be affected by the culture media used to grow the cells. It has been reported that cultivating cells in media with high osmolarity (for example, 350 mOsm) or at lower temperatures (such as 33°C) affects the cell’s virus productivity [25, 26]. The mechanism behind this change however remains ill-defined.

Multi-medium replacements during the cell growth phase and post virus infection were explored to evaluate if infection at higher cell density could further improve the volumetric virus productivity. Figure 4c shows the titers obtained in cultures with 2 medium replacements during the cell growth phase and infected at 6.6 and 7.5 × 10^6^ cells/mL with 3 and 4 medium replacements post virus infection respectively. The virus titers increased to 5.9 and 7.3 × 10^10^ vp/mL, which represents a 4.5 and a 5.6-fold increase when compared to the titer obtained in the culture infected at 1 x 10^6^ cells/mL with medium replacement. These data suggest that the SF-BMAdR-281 clone can grow to higher cell density and cultures infected at higher cell density are able to produce higher titer if sufficient nutrients remain in the culture and inhibitory metabolites are removed from the system. This result once again indicates that nutrient limitation and/or metabolite inhibition are responsible for low maximum cell density and virus productivity. Altogether, these results highlight the potential of perfusion culture process for improving virus productivity.

**Fig. 5:**
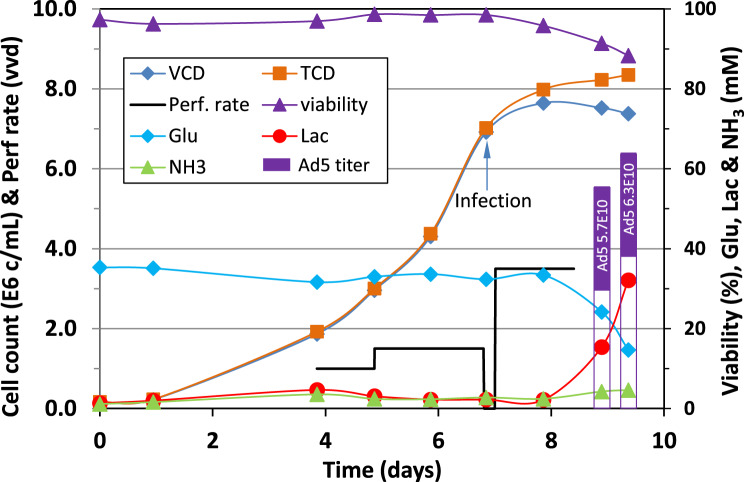
Cell growth and adenovirus production in 3 L single use bioreactor perfusion culture

*Cell growth and Ad5-GFP production in 3 L bioreactor perfusion culture:* The culture condition employed in the shake flask culture, which meant to mimic perfusion and included multiple medium replacements pre- and post- virus infection, was used as basal process parameters in the 3 L bioreactor perfusion culture. In the perfusion culture, the continuous supply of fresh nutrients not only allows the culture to reach high cell density but also provides nutrients required during the virus production phase. As shown in Fig. 5, when the total cell density reached 1.9 × 10^6^ cells/mL or 70 hours after the inoculation, the perfusion was started at 1 VVD for a day and then ramped up to 1.5 VVD for another 48 h to achieve a total cell density of 7.0 × 10^6^ cells/mL before the cells were infected at an MOI of 10. Immediately after the bioreactor was inoculated with the virus, the perfusion was stopped for 3 hours to avoid the viruses being washed out before attaching to the cells. The perfusion was then resumed at 3.5 VVD for another 32 hours. The total cell density reached 8.4 × 10^6^ cells/mL at 60 hpi, while the cell viability declined from 91.4% at 48 hpi to 88% at 60 hpi.

The glucose concentration maintained over 30 mM during perfusion and then declined to 17 mM after the perfusion was stopped for 12 hours. Similarly, lactate concentration was very low ( < 5 mM) when the perfusion was on, and then increased to 36 mM after the perfusion was terminated for 12 hours. The viral titers were 5.7 × 10^10^ and 6.5 × 10^10^ vp/mL at 48 hpi and 60 hpi, respectively, corresponding to a cell specific productivity of 6785 or 7500 vp/cell. The infectious titer of the samples taken at 48 and 60 hpi was respectively 5.7 x 10^9^ and 8.4 x 10^9^ TCID50/mL, which corresponds to 10% and 12.9% of virus particles were infectious. The highest titer achieved in another 3 L bioreactor perfusion culture was 6.14 × 10^10^ vp/mL. This virus productivity is similar to that obtained in the shake flask cultures with multiple medium replacements before and post virus infection (Figure 4c). Both experiments show that removing metabolic waste and replenishing nutrients can significantly improve the virus production. Despite significant improvements in viral titers in perfusion mode, the cell specific virus productivity of 7500 vp/cell is still lower than the 10,700 vp/cell obtained in the shake flask culture process where the medium was replaced before viral infection at 1 × 10^6^ cells/mL. This result could mean that nutrient limitation and/or metabolite inhibition have not been completely resolved even at a high perfusion rate of 3.5 VVD for 32 hours post the virus infection.

Despite some room for improvement, the potential of perfusion culture for increasing volumetric virus productivity is undeniable. The increase in viral titers using perfusion technology, by almost one order of magnitude, has been reported for other viruses and production cells [19, 27]. The perfusion culture is becoming a process of choice for the production of viruses and viral vectors [28], partially due to the limited success of fed-batch culture in the development of high-titer processes for the production of viral vectors and viruses.

## Conclusion

**Fig. 6:**
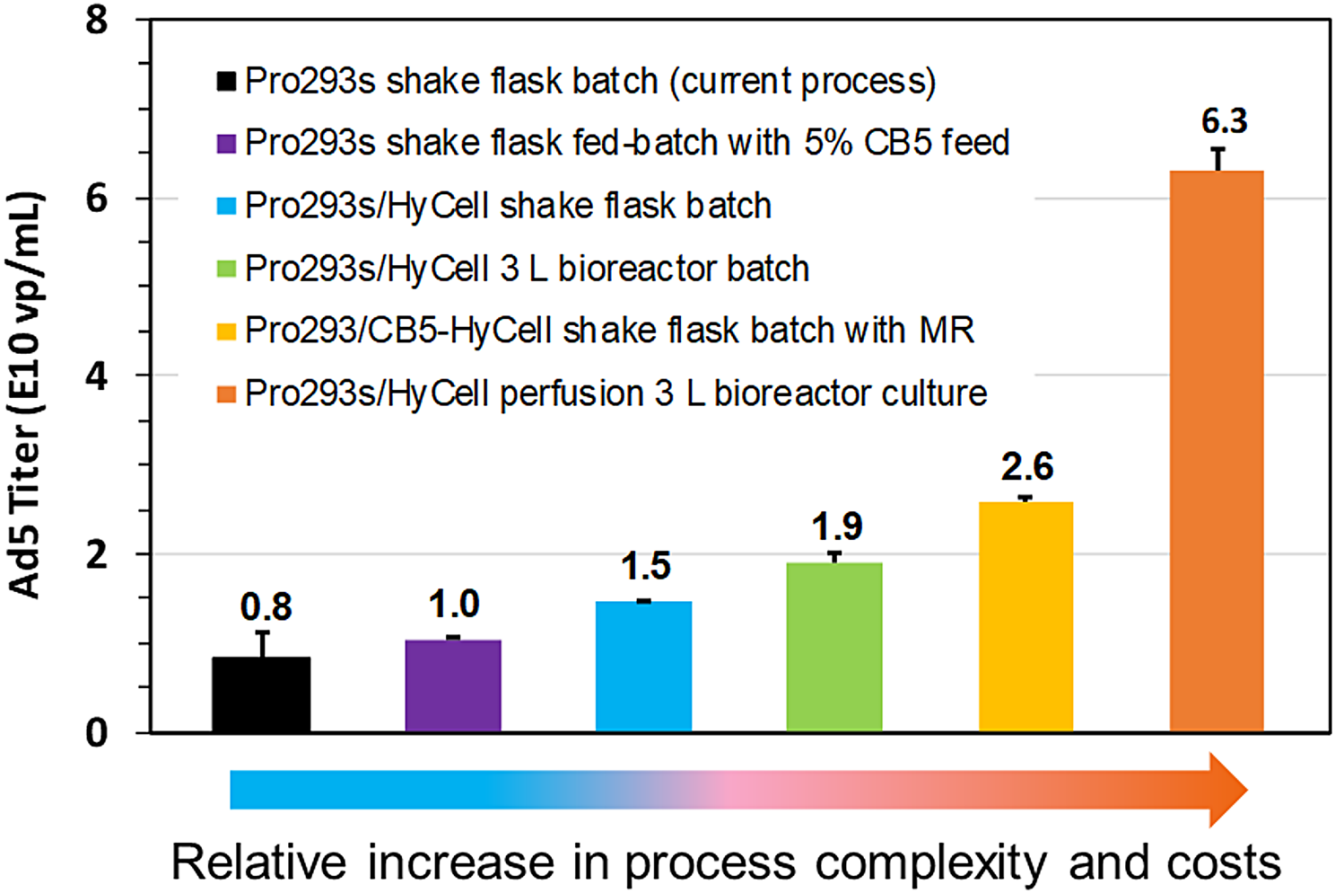
Improvement of adenovirus productivity in suspension A549 culture through process development (MR: medium replacement)

**Fig. 7:**
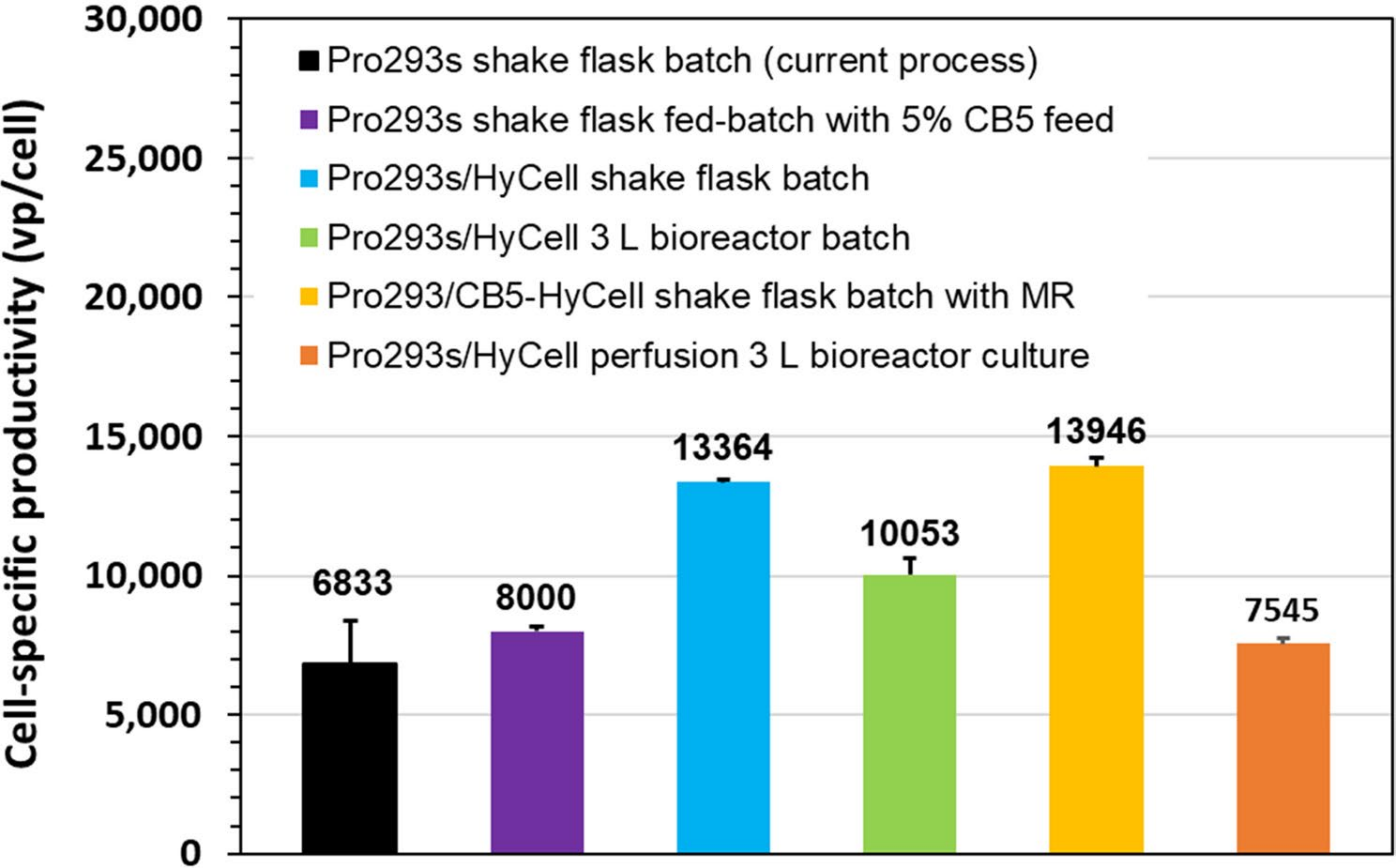
Cell specific virus productivity affected by culture media, processes, and scale up

The relative improvement in virus productivity can be linked to the complexity of the developed culture process as shown in Fig. 6. The batch process utilizing a mixture (1:1) of Pro293s and HyCell appears to be a cost-effective approach to improve the virus productivity, as this process increased the titer by more than 80%, maintained operation simplicity and minimized additional operational costs. Although fed-batch culture does not complexify the process, the addition of feed did not substantially increase the virus production. Medium replacement prior to the virus infection could be a good option to further increase the process productivity as the operation of medium replacement at large-scale production is feasible using alternating tangential flow filtration (ATF) technology. While the perfusion culture engages with increased operational costs such as the volumes of cell culture media, extra equipment, and labor due to the complexity of operations, the major improvement in virus productivity for some viruses is worth the investment in this technology. It is noteworthy that the use of medium mixture and/or medium replacement prior to the virus infection improved the cell specific virus productivity, while the cells lost some productivity when the culture was scaled up to bioreactor as shown in Fig. 7. In summary, this study describes a path to process development to significantly improve the production of RCA-free adenovirus in the SF-MBAdR-281 culture, and provides various options to meet different operational needs in order to meet the increasing demands for viral vectors for gene therapy and therapeutic vaccines.

## Data Availability

Not applicable.
